# Adenovirus-IL-10 relieves chronic rejection after mouse heart transplantation by inhibiting miR-155 and activating SOCS5

**DOI:** 10.7150/ijms.77093

**Published:** 2023-01-09

**Authors:** Gangcheng Kong, Yuqi Chen, Zongtao Liu, Yixuan Wang, Huadong Li, Chao Guo

**Affiliations:** 1 Department of Hepatobiliary and Pancreatic Surgery, the First Affiliated Hospital, School of Medicine, Zhejiang University, Hangzhou, Zhejiang, China.; 2 Department of Cardiovascular Surgery, Union Hospital, Tongji Medical College, Huazhong University of Science and Technology, Wuhan, China.

**Keywords:** Chronic rejection after heart transplantation, Macrophage function, IL-10, SOCS5, microRNA-155, Macrophage

## Abstract

**Objective:** Chronic rejection remains the main factor that influence long-term survival of patients after heart transplantation. Interleukin-10 (IL-10) play critical role in macrophages-mediated transplant immune responses. We investigated the mechanism of IL-10 in macrophage related chronic rejection after mouse heart transplantation.

**Methods:** Mouse heart transplant chronic rejection model was established to evaluate pathological changes in the allograft. Myocardial interstitial fibrosis, apoptosis, and inflammatory factor levels were detected in ad-IL-10-treated mice. The positive iNOS^+^ and Arg-1^+^ expressions, macrophage subset changes, and the proportion of regulatory T-cells (Tregs) and TIGIT^+^ Tregs were quantified by flow. In in vitro experiments, ad-IL-10 was transfected into macrophages followed by detection of apoptosis, phagocytosis, and CD163, CD16/32, and CD206 expression. The expression and relationships between IL-10, miR-155, and SOCS5 were also detected and verified. A rescue experiment was performed to evaluate macrophage function through the combined treatment of ad-IL-10 and overexpression of miR-155.

**Results:** Significantly decreased IL-10 expression in chronic rejection during mouse heart transplantation was observed. Ad-IL-10-treated mice showed decreased pathological injury, perivascular fibrosis, apoptosis, inflammation, and iNOS^+^ and CD16/32^+^ expression, and increased Treg/TIGIT^+^ Treg cell, Arg-1^+^ and CD206^+^ cell proportion. Ad-IL-10-treated macrophages in vitro showed reduced apoptosis, improved phagocytosis, and M2 polarization. Mechanically, IL-10 negatively regulated miR-155 to activate SOCS5. Overexpression of miR-155 reversed IL-10 mediated-positive regulation of macrophage function.

**Conclusion:** IL-10 downregulated miR-155 and activated SOCS5, thereby promoting macrophage M2 polarization to relieve chronic rejection after heart transplantation.

## Introduction

Chronic rejection remains a major problem in organ transplantation 1. Over 50% of patients experience chronic rejection within 5 years following heart transplantation, which causes one-third of deaths and is characterized by cardiovascular lesions, fibrosis, and coronary stenosis 2, 3. Inflammation is the core factor in the acute and chronic rejection of organ transplantation 4. In addition, macrophages are the main cell type involved in infiltration in chronically rejected transplanted hearts 5. Macrophages are important immune cells that are potential targets for anti-rejection therapies in organ transplantation 6. Macrophages exhibit pro-inflammatory phenotypes (M1-type) and anti-inflammatory phenotypes (M2-type) 7. Macrophage phenotypes function in diagnosing myocardial inflammation induced by ongoing rejection in the transplanted heart 8. A study reported that interleukin-10 (IL-10) promotes macrophage phenotype transformation from M1-type to M2-type 9. Therefore, this study aimed to identify a novel IL-10-based therapy for chronic rejection after heart transplantation.

IL-10 is a significant regulator of solid organ transplantation, and IL-10 knockout results in graft dysfunction and death in mice 10. IL-10 inhibits chronic rejection and regulates the immune response in renal allografts, and is characterized by clearly inhibited arterial neointimal hyperplasia, artery occlusion, interstitial fibrosis, and inflammation 11. IL-10 stimulates macrophages to differentiate toward the M2 phenotype, in which macrophages exhibit higher levels of phagocytosis 12. However, the regulatory mechanisms of IL-10 in chronic rejection after heart transplantation through macrophage function remain unclear.

IL-10 regulates cardiac fibrosis through a microRNA (miRNA)-mediated approach 13. miRNAs are important biomarkers for heart transplantation rejection 14. miR-650 is essential for the regulation of B-cell CLL/lymphoma 11B gene expression following acute rejection of renal allografts 15. Moreover, macrophage differentiation is verified to be performed through miRNAs, which bind to mRNA and regulate macrophage phenotype and function 16. miRNAs that modulate macrophage polarization are important in the treatment of inflammation-related diseases 17. However, there are few studies on the mechanism by which IL-10 regulates chronic rejection after heart transplantation through miRNA-mediated regulation of macrophage function.

Therefore, we hypothesized that IL-10 plays an underlying role in chronic rejection after heart transplantation by influencing macrophage function through the miRNA-mRNA system. Consequently, we performed a series of histological and molecular experiments to identify the regulatory mechanism of the IL-10-miRNA-mRNA network involved in macrophage function with the purpose of elucidating novel therapeutic options against chronic rejection after heart transplantation.

## Materials and methods

### Ethical statement

This study was approved and supervised by the Human Ethics Committee of the Tongji Medical College, Huazhong University of Science and Technology. All experiments were conducted in strict accordance with the guidelines of the Animal Care and Use Committee of the Tongji Medical College, Huazhong University of Science and Technology. Animal experiments were conducted based on a minimized number of animals and the least pain on experimental animals.

### Model establishment and animal grouping

C57BL/6 mice (B6, H^-2b^) (females, aged 6-8 weeks) were purchased from Shanghai Laboratory Animal Center, Chinese Academy of Sciences (Shanghai, China) and B6.C-H-2^bm12^KhEg (bm12, H-2^bm12^) mice (females; spontaneous mutation of 1-Ab chain of MHC-II molecule on B6 background) were purchased from The Jackson Laboratory (Bar Harbor, ME, USA) and raised in microisolator cages under specific pathogen-free conditions in the animal facility, with controlled humidity and temperature.

bm12 mice were used as donors and B6 mice were used as recipients to induce chronic rejection in a mouse heart transplantation model based on the instructions of a previous study 18. The mice were allocated to the sham-operated, model, adenovirus-negative control (ad-NC; intraperitoneal injection of 200 mg/kg ad-NC twice weekly from the day before transplantation to the 4^th^ week after transplantation), or ad-IL-10 (intraperitoneal injection of 200 mg/kg ad-IL-10 [Wuhan Biofavor Biotech Co., Ltd., Wuhan, China] twice weekly from the day before transplantation to the 4^th^ week after transplantation) groups 19. Mice in each group were euthanized through intraperitoneal injection of 800 mg/kg pentobarbital sodium (Serva Electrophoresis GmbH, Heidelberg, Germany) in the 2^nd^, 4^th^, and 8^th^ week after heart transplantation. Cardiac tissues from each mouse were collected for histological staining, and cardiac tissue homogenates were collected for real-time quantitative reverse transcription-polymerase chain reaction (qRT-PCR).

### Hematoxylin and eosin (HE) staining

Transplanted hearts were fixed with 10% formaldehyde solution, embedded in paraffin, sectioned into 4 μm pieces, and stained with HE (Solarbio Science & Technology Co., Ltd., Beijing, China). The sections were stained using hematoxylin, differentiation solution, and eosin reagent (Solarbio Science & Technology Co., Ltd., Beijing, China). The sections were later permeabilized with xylene and changes in cell degeneration, necrosis, and inflammatory response of the transplanted heart tissues were observed using an inverted electron microscope (Olympus Optical Co., Ltd., Tokyo, Japan) and photographed. The stained specimens were statistically analyzed using HE.

### Elastica van Gieson (EVG) staining

The prepared heart tissue sections were stained using EVG to observe the stenosis and obstruction of the arterial lumen of the mouse heart. Specific operations were conducted in strict accordance with the manufacturer's instructions (Shanghai Yanjin Biological Co. Ltd., Shanghai, China). The sections were deparaffinized in xylene and rehydrated through graded alcohol. And then, the sections were placed in elastic stain solution for 30 minutes followed by differentiation with 5% ferric chloride solution. The slides were counterstained with Van Gieson solution for 10 seconds, and then were observed under microscope (Olympus Optical Co., Ltd., Tokyo, Japan).

### Masson staining

After dewaxing, the paraffin-embedded sections of the heart tissue were stained with Weigert's iron hematoxylin staining solution for 5 min, differentiated in acidic ethanol for 15 s, returned to blue in Masson's bluish liquid (Guangzhou Vengene Technologies, Guangzhou, China) for 5 min, and stained with Ponceau S solution for 4 min. Thereafter, the sections were washed in 0.2% acetic acid solution, stained with fast green stain for 2 min, and washed in 0.2% acetic acid solution. The sections were then successively placed in 95% ethanol and absolute ethyl alcohol, cleared in xylene, and sealed with neutral gum.

### Immunohistochemical staining

After dewaxing, the sample sections were immersed in a citrate solution at 95-98 °C for 15 min for antigen retrieval. After cooling at room temperature for 20 min, the sections were removed from the antigen retrieval solution, and 3% hydrogen peroxide solution was added to the surface for 25 min, followed by the addition of 5% normal goat serum. After sealing at room temperature for 10 min, the sections were incubated with specific primary antibody working solutions (M1: inducible nitric oxide synthase [iNOS; 1/100, ab15323, Abcam Inc., Cambridge, MA, USA]; M2: arginase 1 [Arg-1; 4 µg/mL, ab92274, Abcam]). All sections were placed in a humidor and incubated overnight at 4 °C, and biotin-labeled immunoglobulin G (IgG) secondary antibody (1/2000, ab205718, Abcam) was added and the sections were incubated at room temperature for 90 min. The sections were then incubated with horseradish peroxidase (HRP)-labeled streptavidin working solution at room temperature for 20 min. Diaminobenzidine chromogenic solution was thenadded for color rendering for 20 min, and hematoxylin was used for counterstaining for approximately 30 s, followed by the addition of alcohol hydrochloride for differentiation for 3 s. The sections were then sealed with neutral gum and observed using a light microscope (Olympus, Japan).

### Terminal deoxynucleotidyl transferase (TdT)-mediated dUTP nick end labeling (TUNEL) staining

The paraffin-embedded sections were dewaxed, hydrated, blocked with H_2_O_2_, detached with protease, and moisturized with buffer. All TUNEL kit operations were performed in strict accordance with the instructions of TUNEL kit (AmyJet Scientific, Inc., Wuhan, China). Apoptotic cells were assessed based on the presence of yellow granules in the nucleus. The apoptotic rate was calculated as follows: five sections were randomly selected from each specimen, four high-power microscopic fields (400 ×) were randomly selected from each section for manual counting, and the apoptotic rate was the average value of the percentage of apoptotic cells in all fields.

### Macrophages isolated culturing and grouping

Mice (B6, 6-8 weeks) were euthanized through intraperitoneal injection of 800 mg/kg pentobarbital sodium, and the marrow liquid from the femurs of mice was collected and centrifuged at 1500 rpm for 10 min to collect the cells. CD14^+^ mononuclear cells were isolated using a CD14^+^ mononuclear cell sorting kit (Miltenyi Biotec GmbH, Bergisch Gladbach, Germany) and cultured in Roswell Park Memorial Institute (RPMI)-1640 medium containing 10% fetal bovine serum (FBS). The cell density was adjusted to 5 × 10^6^ cells/mL, and the cells were inoculated into a 6-well plate at 2 mL inoculation volume per well. After addition of 20 ng/mL macrophage colony-stimulating factor (M-CSF), the cells were placed in an incubator containing 5% CO_2_ at 37 °C for 24 h. The suspended cells were collected and re-incubated in a new 10 cm culture dish (containing 20 ng/mL M-CSF) to cultivate for an additional 4 d. Initial macrophages (M0) from mouse bone marrow were obtained through further culturing for 3 d after changing the medium (containing 20 ng/mL M-CSF). The cells were transfected with ad-NC and ad-IL-10, respectively. Cells transfected with ad-IL-10 were further transfected with mimic NC (Life Technologies Corporation, Gaithersburg, MD, USA) and miR-155 mimic, according to the instructions of the Lipofectamine 3000 kit (Life Technologies). The final concentration of the transfected substance was 50 nM.

### Flow cytometry

Changes in macrophage subsets were detected using flow cytometry. The spleen tissue was placed in cold phosphate-buffered saline (PBS), and a single-cell suspension was obtained using the 200-mesh nylon filtration method. Immune cells were isolated using OptiPrep density gradient centrifugation and resuspended at 1 × 10^6^/mL in RPMI-1640 medium containing 10% FBS. Cluster of differentiation (CD) 68, CD16/32, and CD206 surface staining were used to detect M1- and M2-type macrophages, and a CD68/side-scattered light gate was used to determine the macrophage population and conduct macrophage subset analysis. CD68^+^CD16/32^+^ cells were M1-type macrophages and CD68^+^CD206^+^ cells were M2-type macrophages. The proportion of regulatory T (Treg) and T-cell immunoglobulin and immunoreceptor tyrosine-based inhibitory motif (ITIM) domain (TIGIT)^+^ Treg cells in the spleen of mice was detected using flow cytometry, in strict accordance with the manufacturer's instructions.

Macrophage apoptosis was detected, and 1 × 10^5^ M0 cells in each group were seeded in a 24-well plate for 72 h and then placed in an incubator containing 5% CO_2_ at 37 °C. Simultaneously, 1 μg/mL lipopolysaccharide (LPS) and 20 ng/mL interferon-γ (IFN-γ) (thus, LPS + IFN-γ) were added to the cell medium or not added. Macrophages detached with trypsin were collected, mixed with 200 μL PBS, and gently pipetted to mix. The cells were then transferred to a sterile 1.5 mL Eppendorf tube. The supernatant was discarded after centrifugation. The cells were then fully mixed with 195 μL Annexin V-fluorescein isothiocyanate (V-FITC) (AmyJet Scientific Inc.) binding solution and 5 μL Annexin V-FITC and incubated at room temperature in the dark for 10 min. After centrifugation at 1500 rpm for 10 min, the supernatant was discarded. Subsequently, the cells were fully mixed with 190 μL Annexin V-FITC binding solution and 10 μL propidium iodide (PI) working solution and then placed on ice without light exposure for 30 min. Flow cytometry was performed to determine the rate of apoptosis.

Macrophage phagocytosis was detected and 1 × 10^5^ M0 cells were inoculated into 24-well plates and co-cultured for 72 h with or without LPS + IFN-γ stimulation. The treated macrophages from each group were collected, and the cell density was adjusted to 1 × 10^7^ cells/mL using 10% FBS RPMI-1640 medium containing 100 ng/μL ovalbumin (OVA)-FITC. The cells were incubated at 37 °C for 40 min and washed twice with 1 mL sterile PBS to remove excess OVA-FITC. Flow cytometry was used for cell detection. The proportion of FITC-positive cells was also analyzed. Expression of CD163 (BioLegend, San Diego, CA, USA) on the macrophage surface was detected using flow cytometry in strict accordance with the manufacturer's instructions.

### qRT-PCR detection

Total RNA was extracted from cells and tissues using the TRIzol kit (Invitrogen, Carlsbad, California, USA). After determining RNA concentration and purity, cDNA was synthesized using a reverse transcription kit (Genecopoeia, Rockville, MD, USA). The expression of each gene was detected using the Synergy Brands PCR Master Mix kit (Applied Biosystems, Foster City, CA, USA) with the PCR instrument, and U6 or glyceraldehyde-3-phosphate dehydrogenase as the internal reference. The relative expression of the target genes was calculated using the 2^-ΔΔCt^ method, and primers for the target genes (Table 1) were designed and synthesized by Sangon Biotech Co., Ltd. (Shanghai, China). The experiment was repeated thrice.

### Western blot analysis

Total protein was extracted using adding cell lysis solution, protein concentration was determined using the bicinchoninic acid assay (Invitrogen), and proteins were standardized with reference to GAPDH. Then, 30 μg sample proteins from each group were isolated using 8% sodium dodecyl sulfate-polyacrylamide gel electrophoresis (SDS-PAGE). The proteins were then transferred to polyvinylidene fluoride (PVDF) membranes and blocked using a shaking table at room temperature. After washing, the membranes were incubated with suppressor of cytokine signaling 5 (SOCS5) primary antibody (1/500, ab97283) overnight. HRP-labelled goat anti-mouse secondary antibody was added and the membranes were incubated at room temperature for 4 h, followed by electrochemiluminescence (Beyotime Biotechnology Co., Shanghai, China). Image-Pro Plus software (version 6.0; Media Cybernetics, Bethesda, MD, USA) was used for quantitative analysis.

### Enzyme-linked immunosorbent assay (ELISA)

The levels of related factors (IFN-γ, tumor necrosis factor alpha [TNF-α], Interleukin-1 beta [IL-1β], IL-17A, IL-4, and transforming growth factor-β1 [TGF-β1]) were detected according to the instructions of the ELISA kits (Nanjing SenBeiJia Biological Technology Co., Ltd., Nanjing, China).

### Dual-luciferase reporter gene assay

The sequence of the SOCS5 3'-UTR containing the miR-155 binding site was synthesized. The SOCS5 wild-type (SOCS5-WT) was constructed, and based on the plasmid, the SOCS5 mutant (MUT) plasmid (SOCS5-MUT) was constructed after mutation of the binding site. Subsequently, SOCS5-WT and SOCS5-MUT were mixed with plasmids NC and miR-155, respectively, and transfected into HEK-293T cells (Zhen Shanghai and Shanghai Industrial Co., Ltd., Shanghai, China). Cells were collected and lysed after 48 h transfection. Luciferase activity was detected using a luciferase detection kit (BioVision, San Francisco, CA, USA) and a Glomax20/20 luminometer fluorescence detector (Promega Corp., Madison, Wisconsin, USA).

### Co-immunoprecipitation assay

The cells were fixed with 37% paraformaldehyde, cross-linked at room temperature for 10 min, and the reaction was terminated using glycine. The cell medium was removed and the cells were scraped down for centrifugation at 2 000 rpm at 4 °C for 2 min. Cell precipitate was collected and ultrasonically fragmented after adding SDS lysis buffer. After ultrasound, cells were allocated to the input, IgG NC, or anti-SOCS5 groups. After overnight incubation at 4 °C, protein A magnetic beads were added to each group, and the precipitate was washed after incubation for 2 h. The washed DNA fragments were used for qRT-PCR analysis with the input acting as an internal reference. Relative values were calculated using the 2-^△△Ct^ method.

### Statistical analysis

Statistical Package for the Social Sciences 21.0 (IBM Corp. Armonk, NY, USA) was used for the data analysis. According to the Kolmogorov-Smirnov detection, measurement data were normally distributed and expressed as the mean ± standard deviation. Differences between two groups were evaluated using the *t*-test, and differences among multiple groups were compared using one-way or two-way analysis of variance (ANOVA). Tukey's multiple comparison test was used for pairwise comparisons following ANOVA. The *p* value was calculated using a two-tailed test, and *p* < 0.01 indicated a significant difference.

## Results

### IL-10 expression is downregulated in heart tissues and peritoneal macrophages of mice with chronic rejection after heart transplantation

**Figure 1:**
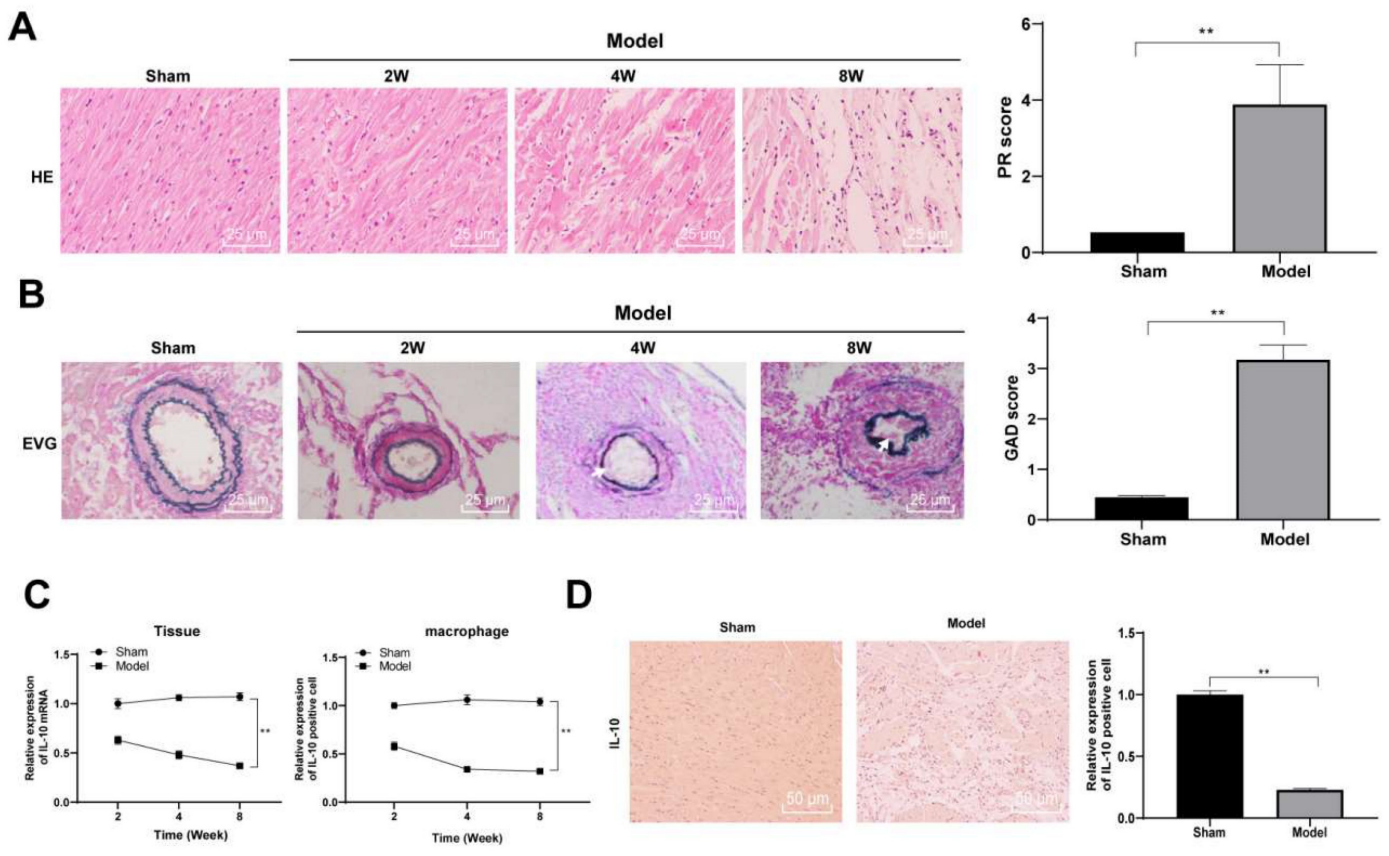
** IL-10 expression is downregulated in heart tissues and peritoneal macrophages of mice in chronic heart transplantation rejection model.** The chronic rejection of heart transplantation model was established with bm12 mice as donors and B6 mice as recipients. HE (A) and EVG staining (B) were used to observe the pathological changes of the hearts of the model mice in the 2^nd^, 4^th^, and 8^th^ week after transplantation, and the pathological injury was quantitatively analyzed. Vascular intimal hyperplasia was marked by arrows. Mice in the sham-operated group were used as controls. Expression of IL-10 in mouse heart tissues and peritoneal macrophages in the 2^nd^, 4^th^, and 8^th^ week after transplantation was evaluated using qRT-PCR (C). IL-10 positive expression in mouse heart tissue was evaluated in the 8^th^ week after transplantation using immunohistochemical staining (D). A/B/D (n = 6), C (n = 3). The data were expressed as mean ± standard deviation and were representative of three independent experiments. Data in panels A/B/D were analyzed using the *t*-test, and data in panel C was analyzed using two-way ANOVA, followed by Tukey's multiple comparison test. ** p < 0.01.

It has been reported that IL-10 improved acute and chronic rejection after lung transplantation in a mouse model 20. To explore the role of IL-10 in a heart transplantation model, we established a chronic rejection model of heart transplantation using bm12 mice as donors and B6 mice as recipients. Vascular lesions in heart grafts were evaluated through pathological changes in the transplanted heart. Mice were euthanized through intraperitoneal injection of 800 mg/kg pentobarbital sodium in the 2^nd^, 4^th^, and 8^th^ week after heart transplantation. Paraffin-embedded sections of mouse heart tissue were prepared. Routine HE and EVG staining were performed to observe the infiltration of inflammatory cells into the heart graft. The heart tissues of mice in the model group exhibited clear histological features of chronic rejection 2 and 4 weeks after transplantation, with specific manifestations of leukocyte infiltration around the blood vessels and in the lumen, worsening vascular stenosis with the course of the disease, a large amount of inflammatory cell infiltration accompanied by cell necrosis and thickening of the vascular mesomembrane in the 8^th^ week after transplantation, suggesting serious heart graft vascular diseases. In contrast, there was no notable inflammatory cell infiltration in the interstitial and perivascular tissues of mice in the sham-operated group and no clear intimal hyperplasia and stenosis of artery vessels (all *p* < 0.01) (Figure 1A/B). Thereafter, the expression of IL-10 mRNA in the heart tissues and peritoneal macrophages of mice was detected in the 2^nd^, 4^th^, and 8^th^ week after transplantation using qRT-PCR. The mRNA expression of IL-10 in the heart tissue and peritoneal macrophages of mice in the model group decreased gradually with transplantation time (all *p* < 0.01) (Figure 1C). IL-10-positive expression in mouse heart tissues was evaluated in the 8^th^ week after transplantation using immunohistochemical staining, which showed that IL-10-positive expression in the heart tissues of model mice was significantly downregulated (*p* < 0.01) (Figure 1D). These results indicate that IL-10 is closely associated with chronic rejection during heart transplantation in mice.

### ad-IL-10 relieves pathological injury and balances inflammatory responses in mice with chronic rejection after heart transplantation

**Figure 2:**
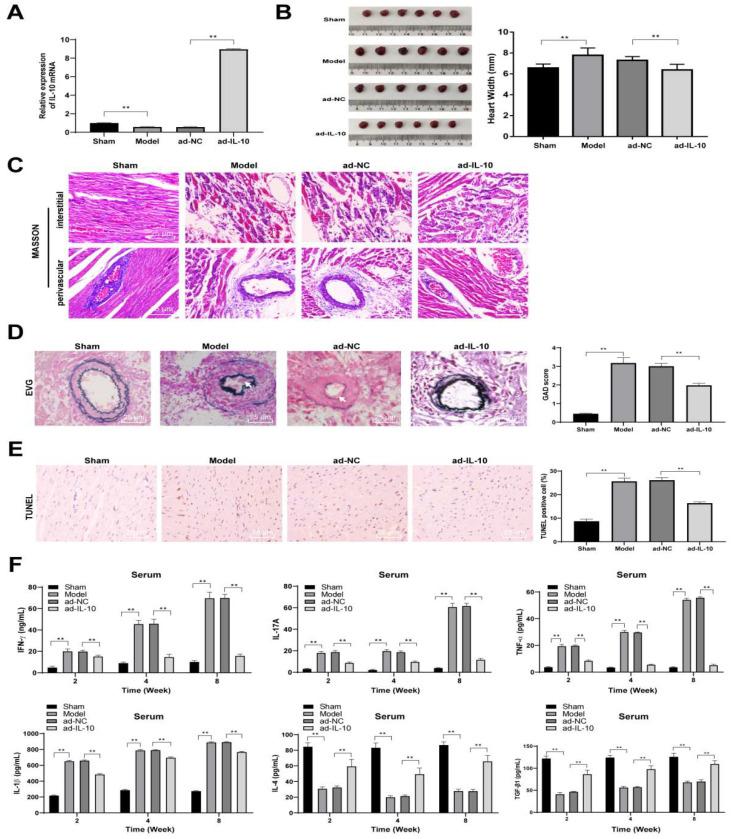
** ad-IL-10 relieves pathological injury of heart transplantation chronic rejection in mice and regulates the balance of pro-inflammatory and anti-inflammatory factors.** ad-IL-10 was injected into the model mice, and expression of IL-10 mRNA was detected using qRT-PCR (A). Texture and hardness of the transplanted heart tissue were observed (B). Fibrosis of the heart tissue was evaluated using Masson staining and the bar graph of heart width messured by vernier caliper (C). Pathological conditions of transplanted heart vessels in each group were analyzed using EVG staining. Vascular intimal hyperplasia was marked by arrows. (D). Positive expression of TUNEL (E) was analyzed quantitatively. Expressions of IFN-γ, TNF-α, IL-1β, IL-17A, IL-4, and TGF-β1 in serum of mice were detected using ELISA (F). A/B/C/D/E (n = 6); F (n = 15). Data were representative of three independent experiments. Data in panels A/D/E were analyzed using one-way ANOVA and data in panel F was analyzed using two-way ANOVA, followed by Tukey's multiple comparison test. ** p < 0.01.

ad-IL-10 was injected into model mice to explore the effect of IL-10 on the chronic rejection of heart transplantation in mice. After injection, the expression of IL-10 mRNA significantly increased in grafts (*p* < 0.01), which was in line with expectations. The texture and hardness of the heart tissues and size of the transplanted heart were observed and recorded. The results of histopathological analysis showed that the transplanted hearts of mice in the model group became hard and dark 8 weeks after transplantation, whereas the hearts injected with ad-IL-10 were smaller in volume, width (*p* < 0.01), softer in texture, and redder in color, and also had a stronger beating intensity and frequency after laparotomy (Figure 2A/B). Masson staining was used to evaluate myocardial fibrosis in the heart tissues of mice in each group, and showed that the degree of myocardial fibrosis in the interstitial and perivascular tissues of mice in the ad-IL-10 group was significantly reduced (Figure 2C). EVG staining showed that the degree of vascular occlusion in the ad-IL-10 group was significantly lower than that in the model group (all *p* < 0.01) (Figure 2D). After ad-IL-10 injection, the TUNEL-positive rate in the heart tissues of mice decreased significantly (all *p* < 0.01) (Figure 2E). ELISA showed that after ad-IL-10 injection, the levels of pro-inflammatory factors (IFN-γ, TNF-α, IL-1β, and IL-17A) in the serum of mice were significantly lower than those in the model group, whereas the levels of anti-inflammatory factors (IL-4 and TGF-β1) were significantly higher (all *p* < 0.01) (Figure 2F). These results show that ad-IL-10 notably reduces the incidence of cardiovascular disease and interstitial fibrosis and improves the function of the transplanted heart, thus alleviating chronic heart transplant rejection.

### ad-IL-10 promotes M2 polarization of macrophages in chronic rejection after mouse heart transplantation

**Figure 3:**
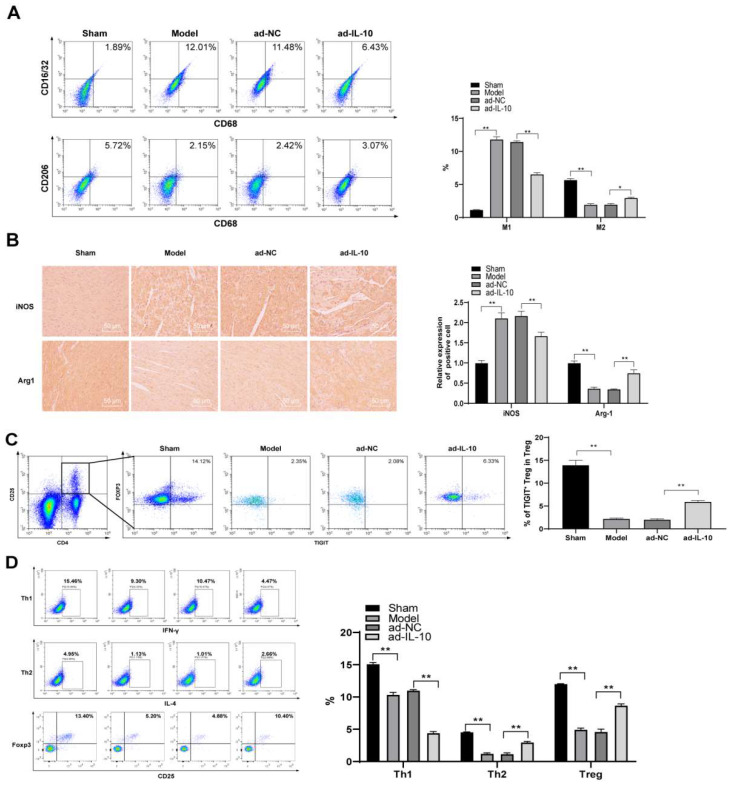
** ad-IL-10 promotes macrophage M2 polarization in chronic rejection after mouse heart transplantation.** ad-IL-10 was injected into the model mice, and the changes of macrophage subsets in the mice spleen were detected using flow cytometry (A). Positive expressions of iNOS and Arg-1 in the mice heart tissue were evaluated using immunohistochemistry (B) and analyzed quantitatively. Treg and TIGIT+ Treg proportions were detected using flow cytometry (C-D). A/B/C/D (n = 6). Data were representative of three independent experiments. Data in panel D was analyzed using one-way ANOVA and data in panels A/B/C were analyzed using two-way ANOVA, followed by Tukey's multiple comparison test. ** *p* < 0.01.

Macrophages are important immune cells, and changes in their function during rejection can better reflect the recovery of patients after transplantation.^21^ Therefore, changes in macrophage subsets in the spleen of mice were detected using flow cytometry. The results showed that the number of CD68^+^CD16/32^+^ (M1-type) cells in the spleen of mice in the model group increased significantly, the proportion of CD68^+^CD206^+^ (M2-type) cells decreased significantly, and ad-IL-10 treatment promoted the differentiation of macrophages into the M2-type (all *p* < 0.01) (Figure 3A). Expression of iNOS^+^ (M1-type) and Arg-1^+^ (M2-type) in the heart tissues of mice in each group was evaluated by immunohistochemistry. After treatment with ad-IL-10, more Arg-1^+^ cells infiltrated the perivascular heart tissues of mice, and only a small number of iNOS^+^ cells appeared (all *p* < 0.01) (Figure 3B). Tregs play an important role in slowing down transplantation rejection and inducing transplantation tolerance. Therefore, the Treg and TIGIT^+^ Treg cell proportions were detected using flow cytometry. The proportions of Treg and TIGIT^+^ Treg cells in the spleens of mice treated with ad-IL-10 were significantly higher than those in the model group (all *p* < 0.01) (Figure 3C/D). The above results showed that ad-IL-10 promoted M2 polarization of mouse macrophages during chronic rejection after heart transplantation.

### ad-IL-10 enhances macrophage phagocytosis and promotes macrophage differentiation to the M2 type

**Figure 4:**
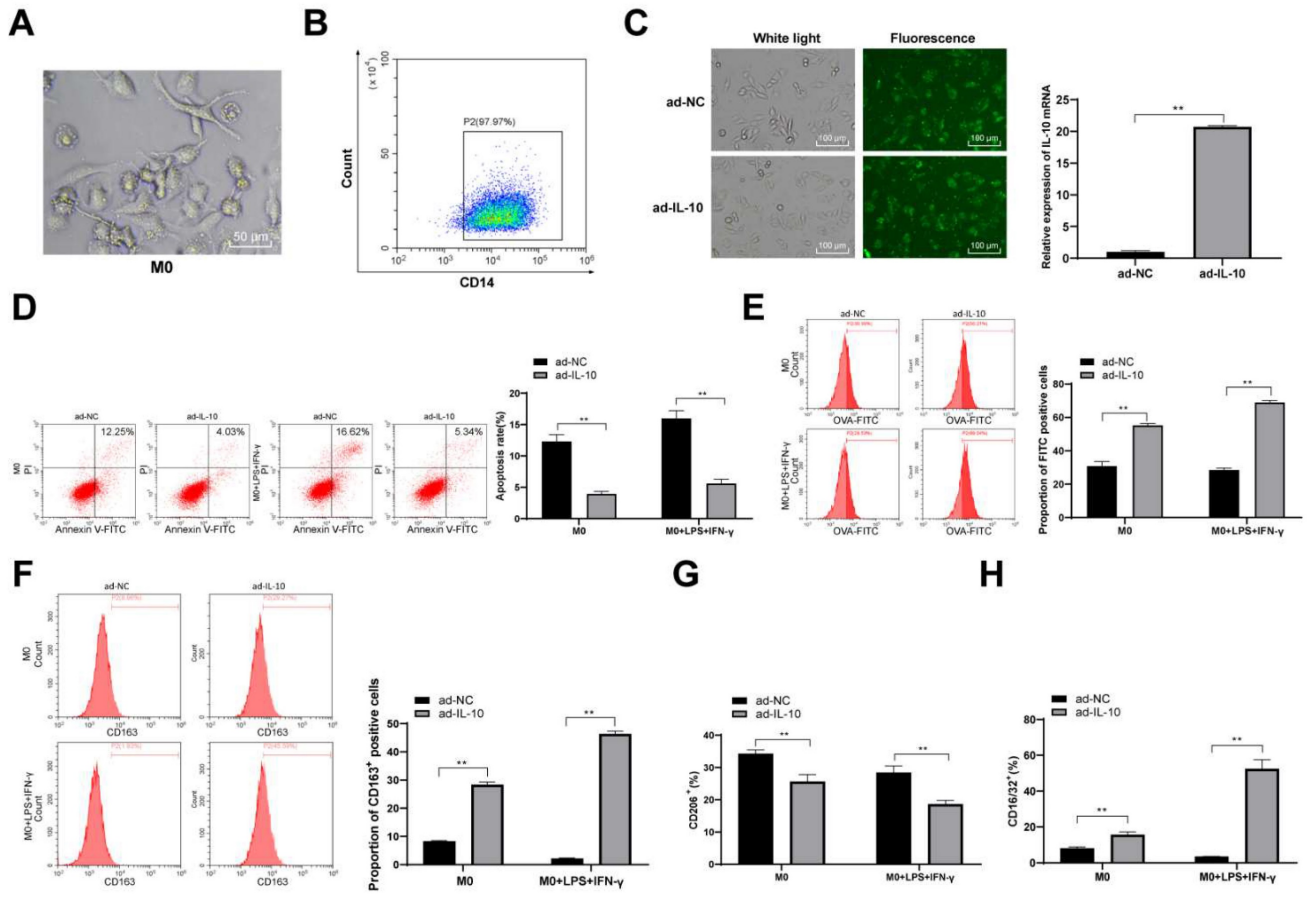
** ad-IL-10 enhances macrophage phagocytosis and promotes macrophage differentiation to M2 type.** Cell morphology was observed by a microscope (A), and flow cytometry (B) was used to detect the purity of isolated macrophages. ad-IL-10 was transfected into macrophages, and the transfection efficiency was detected using qRT-PCR (C). Macrophages treated with ad-IL-10 were transfected with miR-155 mimic, and then flow cytometry was used to detect apoptosis (D), phagocytosis (E), CD163^+^ expression (F), CD16/32 (M1) expression (G), and CD206 (M2) expression (H). Data were presented as means ± SD (n = 6) and were representative of three independent experiments. Data in panel C was analyzed using the *t*-test, and data in the rest panels were analyzed using two-way ANOVA, followed by Tukey's multiple comparison test. ** *p* < 0.01.

To further confirm the regulatory effect of IL-10 on macrophage function, mouse peritoneal macrophages were isolated and cultured, and the results of fluorescence-activated cell sorting revealed that the purity of the isolated mouse peritoneal macrophages reached 96% (Figure 4A). Thereafter, ad-IL-10 was transfected into mouse peritoneal macrophages, and fluorescence microscopy showed that the transfection efficiency was > 80% (*p* < 0.01) (Figure 4B/C). Macrophage apoptosis was detected in each group, and the results revealed that the apoptosis rate of macrophages treated with ad-IL-10 decreased significantly (Figure 4D). To detect the effect of IL-10 on macrophage phagocytosis, macrophages were stimulated with LPS and IFN-γ for 24 h and observed using flow cytometry. The phagocytosis of macrophages treated with ad-IL-10 was significantly enhanced whether LPS + IFN-γ stimulation was administered or not (all *p* < 0.01) (Figure 4E). CD163 is closely associated with macrophage phagocytosis. The expression of CD163 on the macrophage surface in each group was further detected using flow cytometry, and the results showed that the number of CD163^+^ cells was noticeably increased after ad-IL-10 treatment, regardless of LPS + IFN-γ stimulation (Figure 4F). The expression of CD16/32 (M1) and CD206 (M2) on the macrophage surface in each treatment group was detected using flow cytometry, and the results showed that ad-IL-10 promoted the differentiation of macrophages into the M2-type (all *p* < 0.01) (Figure 4G/H).

### IL-10 downregulates miR-155 to promote SOCS5 expression

**Figure 5:**
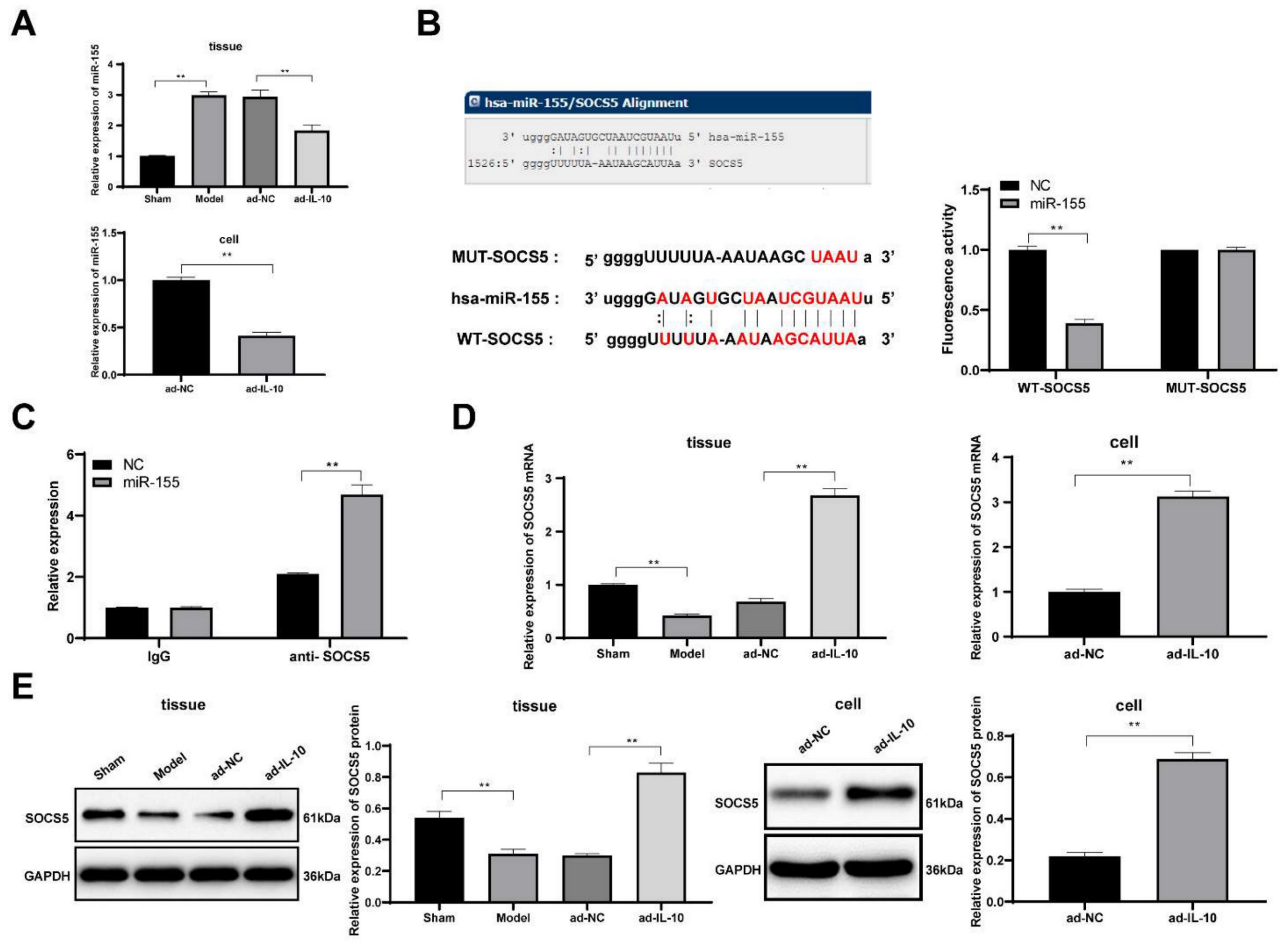
** IL-10 activates SOCS5 expression by downregulating miR-155.** Expression of miR-155 in mouse heart tissue and macrophages were detected using qRT-PCR (A). It was predicted that there was a targeted binding relationship between miR-155 and SOCS5 through the biological website, which was verified using dual-luciferase reporter gene assay (B) and co-immunoprecipitation assay (C). Expressions of SOCS5 mRNA and protein were detected using qRT-PCR and western blot analysis (D-E). Data were presented as means ± SD (n = 6) and were representative of three independent experiments. Data in panels A/D/E were analyzed using one-way ANOVA, and data in panels B/C were analyzed using two-way ANOVA, followed by Tukey's multiple comparison test. ** *p* < 0.01.

These studies confirmed that IL-10 reduced the chronic rejection of mouse heart transplantation and promoted the differentiation of mouse peritoneal macrophages into the M2-type. However, the underlying mechanism of action of IL-10 remains unclear. miR-155 plays an important role in macrophage polarization process of macrophages 22. Cytokines can affect the development of diseases by regulating miRNAs 23. Therefore, we hypothesized that IL-10 may affect macrophage function by regulating miR-155 expression. The expression of miR-155 in mouse heart tissues and macrophages was detected using qRT-PCR, and the results showed that ad-IL-10 resulted in decreased miR-155 expression in mouse heart tissues and macrophages (all *p* < 0.01) (Figure 5A), indicating that IL-10 negatively regulates miR-155. It was predicted that there was a targeted binding relationship between miR-155 and the immune-related gene SOCS5 through the biological website, which was verified using a dual-luciferase reporter gene assay and co-immunoprecipitation assay (all *p* < 0.01) (Figure 5B/C). Additionally, qRT-PCR and western blot analysis showed that ad-IL-10 led to notably increased SOCS5 expression in mouse heart tissues and macrophages (all *p* < 0.01) (Figure 5D/E). The above results show that IL-10 may activate SOCS5 expression by downregulating miR-155, thereby playing a regulatory role in macrophage function in chronic rejection after heart transplantation.

### Overexpression of miR-155 reverses the positive regulation of IL-10 on macrophage function

**Figure 6:**
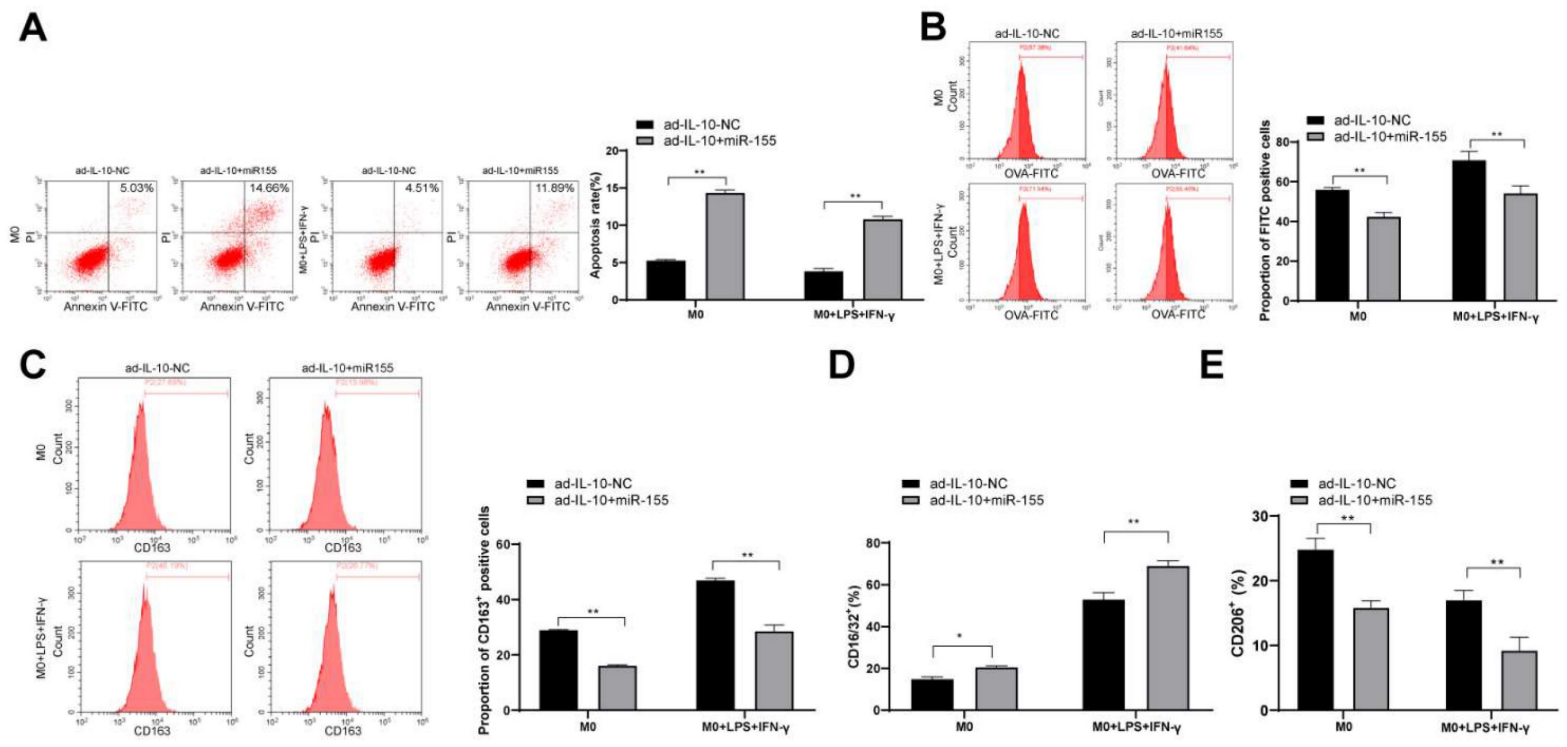
** Overexpression of miR-155 reverses the positive regulation of IL-10 on macrophage function.** Overexpressed miR-155 was transfected into the macrophages treated with ad-IL-10. Flow cytometry was used to detect apoptosis, phagocytosis, CD163^+^ expression, CD16/32 (M1) expression, and CD206 (M2) expression (A-E). Three independent repeated tests were conducted. The data were expressed as mean ± standard deviation and were representative of three independent experiments. The data were analyzed using two-way ANOVA, followed by Tukey's multiple comparison test. ** p < 0.01.

To further confirm that IL-10 affects macrophage function by targeting miR-155, overexpressed miR-155 was transfected into macrophages treated with ad-IL-10. The effects of the combination of ad-IL-10 and overexpression of miR-155 on the phagocytic ability and polarization of macrophages were evaluated. The results showed that overexpression of miR-155 inhibited the phagocytic ability and differentiation M2-type of macrophages treated with ad-IL-10 (all *p* < 0.01) (Figure 6A/E), indicating that miR-155 overexpression reversed the positive regulation of IL-10 on macrophage function.

## Discussion

IL-10 is involved in the inhibition of immunopathology and graft failure in organ transplantation 10. Macrophages are reported to play a significant role in the injury and repair of chronic rejection in transplanted hearts 24. In this study, we found that IL-10 was downregulated in macrophages of mice with chronic rejection after heart transplantation, and ad-IL-10 relieved pathological injury in mice with chronic rejection after heart transplantation and promoted macrophage differentiation toward the M2-type through the IL-10/miR-155/SOCS5 axis.

The current study found that IL-10 was downregulated in the heart tissues and peritoneal macrophages of mice with chronic rejection after heart transplantation. Chronic rejection after heart transplantation induces significant vascular stenosis and fibrosis 25. Our results showed that ad-IL-10 significantly reduced interstitial and perivascular fibrosis, vascular stenosis, and the TUNEL-positive rate in the heart tissues of mice after heart transplantation, thus relieving chronic rejection. IL-10 is also involved in the development of vascular stenosis 26. IL-10 slows tissue fibrosis and chronic rejection 27. Organ transplant rejection is associated with inflammation 28. Our results showed that IL-10 overexpression decreased the levels of pro-inflammatory factors (IFN-γ, TNF-α, IL-1β, and IL-17A) and increased the levels of anti-inflammatory factors (IL-4 and TGF-β1), indicating alleviated inflammatory responses. IL-10 induces macrophages toward a non-inflammatory state and thus regulates inflammatory conditions 29. The results suggested that ad-IL-10 attenuated pathological injury and inflammation of the transplanted heart, thereby relieving chronic rejection after heart transplantation.

Besides IL-10, several potential cytokines may participate in the procedure of rejection after transplantation. High IL-21 level has been found to maintain immune homeostasis after transplantation.^30^ IL-21 promoted the polarization of primary alveolar macrophages toward the M2 phenotype after lung transplantation.^31^ In heart transplantation model, allografts survival could be significantly prolonged in the IL-21R deficient recipients.^32^ Recipients showed significantly higher IFN-γ and significantly lower IL-4 plasma levels late post-transplantation.^33^ IL-3 and IL-4 triggered chronic rejection of cardiac allografts by activation of infiltrating basophils.^1^, ^34^ IL-9 was involved in eosinophil infiltration of cardiac allografts and the Th2 alloimmune response in CD8-deficient mice was associated with the accumulation of IL-9 mRNA in the rejected graft.^35^ Moreover, low-dose IL-2 could delay rejection in a murine model of chronic cardiac allograft rejection,^36^ while IL-17 promoted the pathogenesis of chronic allograft rejection.^37^ After injection of Ad-IL-10 in our study, the levels of pro-inflammatory factors (IFN-γ, TNF-α, IL-1β, and IL-17A) in the serum of mice were significantly lower than those in the model group, whereas the levels of anti-inflammatory factors (IL-4 and TGF-β1) were significantly higher. The mechanisms remain uncertain and the superposed effect provide the following insights for future research.

Macrophages play a critical role in the inflammatory response and innate immunity by altering their phenotype from pro-inflammatory (M1) to anti-inflammatory (M2) 38. M1-type macrophages are characterized by CD16/32 and iNOS expression, whereas M2-type macrophages are characterized by CD206, IL-10, and Arg-1 expression 28. Tregs are critical for combating inflammation and autoimmunity 39. High expression of CD163 is associated with increased macrophage phagocytosis 40. In this study, the results revealed that ad-IL-10 caused higher expression of CD206^+^, Arg-1^+^, TIGIT^+^ Treg cells, and CD163^+^ in the macrophages of mice with chronic rejection after heart transplantation, suggesting that ad-IL-10 promoted the differentiation of macrophages into the M2-type and enhanced macrophage phagocytosis. IL-10 suppressed pro-inflammatory M1-type macrophage activation 29. IL-10 expression is upregulated in TIGIT^+^ cells, and cerebral Treg cells restrict macrophage differentiation toward the M1-type 41, 42. High IL-10 expression contributes to the relief of chronic rejection after heart transplantation by regenerating macrophages toward M2 polarization 2. IL-10 signaling is involved in regulating macrophage phagocytosis 43. These results revealed that IL-10 promoted macrophage M2 polarization and enhanced macrophage phagocytosis in mice with chronic rejection during heart transplantation.

Regulatory B cells participated in antibody-mediated rejection.^44^, ^45^ However, when B6 mice underwent heart transplantation with Bm12 hearts, donor-specific antibody won't be made. In an MHC minor mismatched transplantation model, independently of macrophages, Tregs level could regulate chronic murine allograft rejection.^46^ After in vitro expansion and reinfusion back, Tregs induced long-term tolerance cardiac allografts.^47^ In our study, the proportions of Treg and TIGIT^+^ Treg cells in the spleens of mice treated with ad-IL-10 were significantly higher than those in the model group. Since immunity system is complex and crisscross, changes in Tregs may be influenced by macrophages or vice versa. The detailed mechanism needs further investigation in the future.

These studies confirmed that IL-10 reduced chronic rejection after heart transplantation, promoted macrophage differentiation into the M2-type, and enhanced macrophage phagocytosis. However, the downstream regulatory mechanism of IL-10 remains unclear. Adenosine deaminase acting on double-stranded RNA 1 (ADAR1) inhibits allogeneic graft rejection by inducing macrophage M2 polarization through the ADAR1/miR-21/forkhead box protein O1/IL-10 axis 48. In this study, IL-10 downregulated miR-155 expression, thereby playing a positive role in macrophage function in chronic rejection during heart transplantation. IL-10 was reported to negatively regulate miR-155 to relieve macrophages inflammation and alleviate intestinal immune injury 49, 50. Moreover, miR-155 overexpression reversed the positive effects of IL-10 on macrophage function. miR-155 has been reported as a potential target for heart rejection treatment 51. In this study, the targeted binding relationship between miR-155 and the immune-related gene, SOCS5, was verified. Suppressed miR-155 positively regulates the immune function of Kupffer cells and prolongs liver allograft survival 52. SOCS5 has potent immune functions that are closely associated with IL-10 mRNA expression in mixed lymphocyte reactions, which is an organ transplant rejection in vitro 53, 54. Downregulated SOCS5 of in liver cell is related to the M1 polarization of macrophages 55. The above results indicate that IL-10 improved mouse chronic rejection after heart transplantation and promoted macrophage differentiation toward the M2-type through the IL-10/ miR-155/ SOCS5 axis.

There are several major limitations of our present study. For other relative inflammatory cytokines, an intensive study still needs to be conducted in the future. Through Ad-IL-10 intraperitoneal injection, it is hard to demonstrate our view in tissue-specific level since its low targeting. lesion degree of tissues and vessels need refinement followed over time. The mode of interaction between IL-10 and miR-155, the mechanisms of interaction between macrophage and other immunity cells and by which IL-10 acts on Tregs remains unknown remain unknown. Future research should be undertaken to explore.

Overall, our study supports that IL-10 improves pathological injury and inflammation, promotes macrophage differentiation into the M2-type, and enhances macrophage phagocytosis in mice with chronic rejection after heart transplantation by activating SOCS5 and downregulating miR-155. These results reveal a novel IL-10-based therapy for chronic rejection after heart transplantation, and blocking the IL-10/miR-155/SOCS5 axis is a promising therapeutic approach for the treatment of chronic rejection after heart transplantation. Although the present study provides therapeutic value for treating chronic rejection after heart transplantation, the experimental results and clinical applications need to be further verified.

## Figures and Tables

**Table 1:** Primer sequences for RT-qPCR

Primer	Sequence
miR-155	F: GGGGTTAATGCTAATCGTGA
	R: CAGTGCGTGTCGTGGAGT
U6	F: CTCGCTTCGGCAGCACA
	R: AACGCTTCACGAATTTGCGT
IL-10	F: GCATACGCATTATGTGGGCA
	R: CCTTGCACCTTCTCCGACTT
SOCS5	F: ATTGATGGGCTCCCTCTACCC
	R: TGCCTTGACTGGTTCTCGTTCC
GAPDH	F: CAAGGTCATCCATGACAACTTTG
	R: GTCCACCACCCTGTTGCTGTAG

## Abbreviations

ADAR1: adenosine deaminase acting on double-stranded RNA 1
SOCS: suppressor of cytokine signaling
bm12: B6.C-H-2^bm12^KhEg
ad-NC: adenovirus-negative control
HE: hematoxylin and eosin
EVG: Elastica van Gieson
iNOS: inducible nitric oxide synthase
Arg-1: arginase 1
HRP: horseradish peroxidase
TUNEL: terminal deoxynucleotidyl transferase (TdT)-mediated dUTP nick end labeling
TIGIT: T-cell immunoglobulin and immunoreceptor tyrosine-based inhibitory motif (ITIM) domain
LPS: lipopolysaccharide
OVA: ovalbumin
SDS-PAGE: sodium dodecyl sulfate-polyacrylamide gel electrophoresis
ELISA: enzyme-linked immunosorbent assay
MUT: mutant
